# Chemical genetics in drug discovery

**DOI:** 10.1016/j.coisb.2017.05.020

**Published:** 2017-08

**Authors:** Elisabetta Cacace, George Kritikos, Athanasios Typas

**Affiliations:** European Molecular Biology Laboratory, Genome Biology Unit, Heidelberg, Germany

**Keywords:** High-throughput screening, Drug target, Drug interactions, Genomics, Drug resistance

## Abstract

Chemical-genetic approaches are based on measuring the cellular outcome of combining genetic and chemical perturbations in large-numbers in tandem. In these approaches the contribution of every gene to the fitness of an organism is measured upon exposure to different chemicals. Current technological advances enable the application of chemical genetics to almost any organism and at an unprecedented throughput. Here we review the underlying concepts behind chemical genetics, present its different vignettes and illustrate how such approaches can propel drug discovery.

## Highlights

• Quantitative reverse genetics approaches can delineate the cellular function of a drug, revealing its targets and its path in and out of the cell.
• A major such approach, chemical genetics, involve the systematic assessing of gene–drug interactions.
• Chemical genetics can provide insights into the level of cross-resistance between drugs and their underlying mechanistic basis.
• Chemical genetics can serve as basis for predicting drug behaviors and drug–drug interactions.

## Introduction

**Figure 1:**
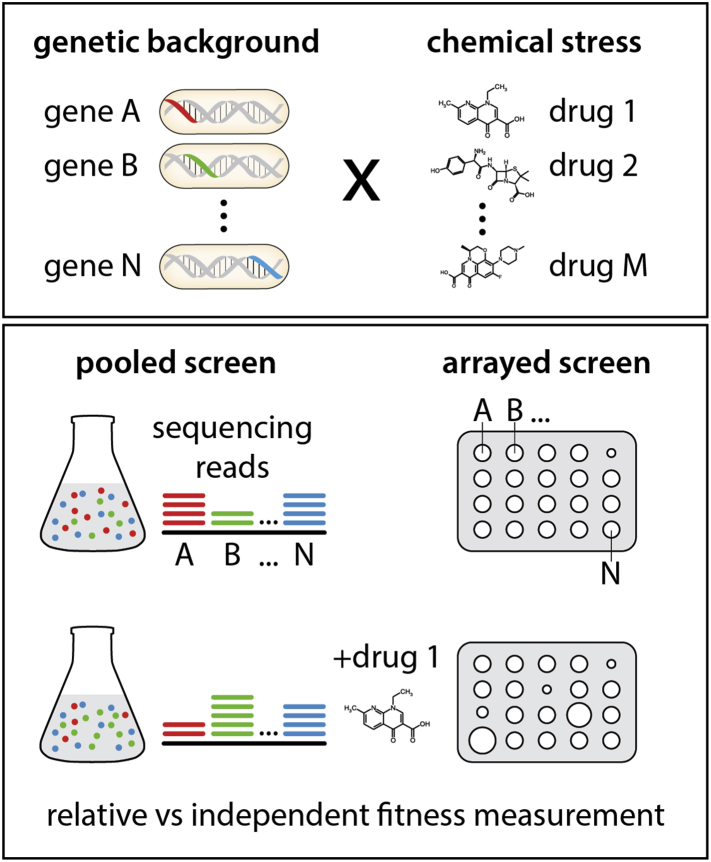
**Basic concepts and approaches in chemical genetics**. Chemical-genetic approaches are based on the combination of genetic and chemical perturbations. The fitness of genome-wide libraries of gain-of-function and loss-of-function mutations is assessed upon exposure to large numbers of drugs. Mutant libraries can be pooled or arrayed. In pooled screens barcoded mutants compete among each other after exposure to a certain drug, and their relative abundance is measured by barcode sequencing. In arrayed screens mutants are ordered and their fitness or additional macroscopic phenotypes can be assessed in an independent fashion.

*Chemical genomics* and *chemical genetics* are often used interchangeably in literature. *Chemical genomics* is a broader umbrella term describing different types of large-scale *in vivo* approaches used in drug discovery, including *chemical genetics* but also large-scale screening of compound libraries for bioactivity against a specific cellular target/phenotype. In contrast, the term *chemical genetics* refers specifically to the systematic assessment of the impact of genetic variance on the activity of a drug (Figure 1). Chemical genetics was pioneered in microbes ∗∗1, ∗∗2, 3, ∗∗4, but is now increasingly applied in human cell lines ∗5, ∗6. The focus of this review will remain on chemical-genetic approaches in microbes, briefly introducing the enabling tools and highlighting the benefits of these applications to drug discovery – identification of Mode-of-Action (MoA), mapping of uptake and efflux routes, revealing of resistance mechanisms and understanding of interactions with other drugs.

## Basis of chemical genetics

High-throughput reverse genetics approaches, such as chemical genetics, have been propelled by the revolution in our ability to generate and track genetic variation for large population numbers. Genetic variation used in such screens comes in many flavors, ranging from controlled to natural. In its most powerful iteration, genome-wide libraries containing mutants of each gene in the chromosome are profiled for changes in the effect of a drug to the organism. Such libraries consist of loss-of-function (LOF; knockout, knockdown) or gain-of-function (GOF; overexpression) mutations and can be arrayed or pooled (Figure 1). In the past decade mutant libraries have been constructed in a plethora of bacteria and fungi [7]. More recently, our proficiency in generating genome-wide pooled mutant libraries [8] and de-convoluting via multiplexing sequencing approaches 9, 10 has brought us to a stage where libraries can be created for almost any microorganism [11]. Although natural genetic variation is frequently used in chemical genetics in human cell lines ∗5, ∗6, ∗12, this unlimited resource has only been recently explored in bacteria [13], leading to similar abilities to delineate drug function as ordered libraries.

To perform reverse genetics in large-scale, creating systematic genetic variance is not enough; one needs to also quantitatively phenotype these populations. Barcoding approaches, pioneered in bacteria [14] and perfected in yeast [15], together with advances in sequencing technologies, have allowed for tracking the relative abundance, and thus the fitness of individual mutants in pooled libraries with unprecedented throughput and dynamic ranges ∗16, ∗17. Thereby differences in relative abundances of mutants in the presence and absence of a drug can reveal genes required or being detrimental for the organism to withstand the drug's cytotoxic effects ∗∗11, 18. Similarly, experimental automation and image processing software 19, ∗20 allows for chemical genetics in arrayed libraries, where hundreds to thousands of mutants are profiled on the same plate ∗∗2, ∗∗4, 21. In arrayed formats, the effects of drugs can be assessed by additional macroscopic phenotypes other than growth, including developmental processes, such as biofilm formation and sporulation, DNA uptake, or cell lysis ∗20, 22, 23. Although microbial chemical-genetic screens have concentrated on measuring bulk phenotypes, quantifying single-cell phenotypes and population behaviors across mutant libraries is also possible with current advances in high-throughput microscopy 24, ∗25. In such cases, cell markers and classifiers of drug responses can provide further insights into the biological activity of the drug in the cell [26]. Single-cell readouts and multi-parametric phenotyping analysis are more common in chemical genetics in human cell lines [12].

## Chemical genetics in MoA identification

**Figure 2:**
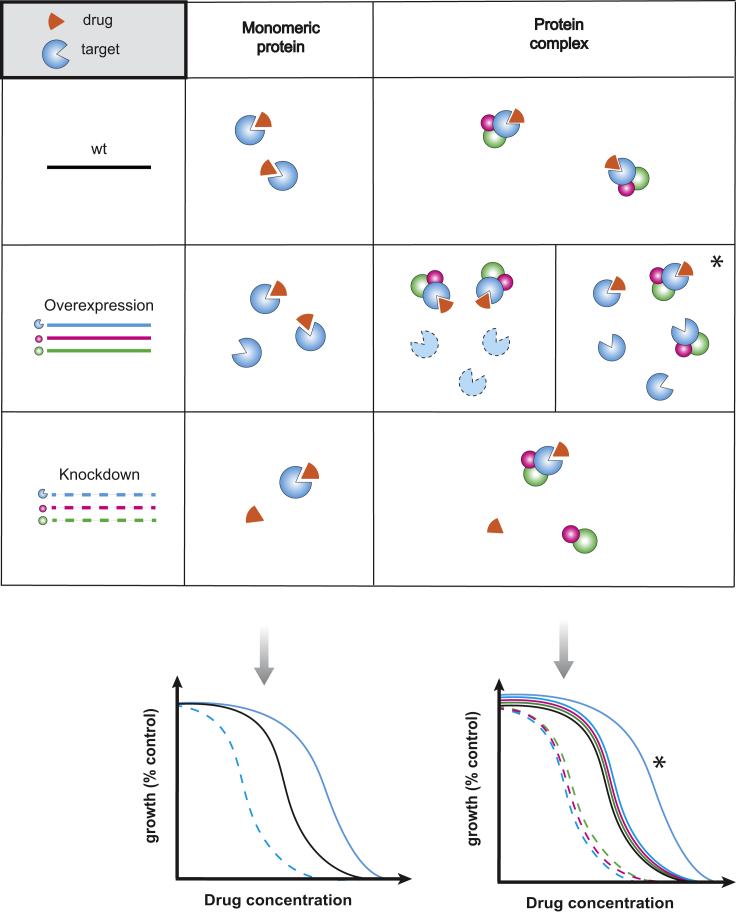
**Gene-dosage perturbations reveal drug target**. Gene dosage perturbations lead to different insights on a drug MoA depending on the nature of the drug target. In the case of a monomeric protein target, its overexpression determines a right-shift in the growth inhibition curve of the drug (i.e. higher drug concentrations are needed to produce the same growth inhibition). However, if the drug target belongs to a protein complex, a curve shift in comparison with the wildtype one is evident only if the drug can bind to the subunit alone and if the subunit is present/functionally active in the absence of its partners (*). If the protein is a functional target or simple present only as part of the complex, then overexpression does not yield any evident change in the effect of the drug. Overexpression of complex co-members does not change the drug’s inhibition curve. Knockdown perturbations can cause detectable changes in the drug growth inhibition curve (left-shift), both in the case of monomeric and of protein complexes targets. In the case of protein complexes, both the target protein and its complex co-members are shifted.

There are two main ways that chemical genetics can be used to map drug targets. First by using libraries in which the levels of essential genes, the usual target of drugs, can be modulated. In this case, when the target gene(s) is down-regulated the cell often becomes more sensitive to the drug (as less drug is required for titrating the cellular target), and the opposite holds true for target gene overexpression (Figure 2). For diploid organisms, heterozygous deletion mutant libraries can be used to reduce the dose of essential genes. Such screens, dubbed as HaploInsufficiency Profiling (HIP), were the first to be used to successfully map drug cellular targets in yeast 3, 27, ∗28. As bacteria are haploids, increasing gene levels is technically simpler. Thus, target overexpression has been repeatedly used to identify the target of new drugs ∗28, 29, 30. Recently, with the advance of CRISPR-based technologies, CRISPRi libraries of essential genes have been constructed in different bacteria ∗∗31, 32 and used for identifying drug targets [31]. Compared to overexpression approaches, knockdown libraries of essential genes have the advantage of being better tailored for capturing the cellular target when this is part of a protein complex (Figure 2). Nevertheless, both approaches have caveats, as genes conferring directly/indirectly resistance to the drug are also detected as hits, and steady-state experiments after induction or down-regulation of an essential gene may result in more drastic effects in the cellular network than just changes in the levels of the gene targeted. Such caveats can be partially overcome by combining results of increased and decreased gene dosage [33] and by more generally titrating gene dosage, or by checking dynamic responses after modulating the levels of essential genes. Nevertheless, more complex drug-target relationships may remain unresolved by simply changing the target levels [34]. Knockdown and overexpression approaches are now starting to be used to identify drug targets in human cells lines 35, 36.

A second way to infer the drug target from chemical genetics data is by comparing drug signatures ∗∗2, ∗∗4. A drug signature comprises the compiled quantitative fitness scores for each mutant within a genome-wide deletion library (all non-essential genes) in the presence of the drug. Drugs with similar signatures are likely to share cellular targets and/or cytotoxicity mechanisms ∗∗2, ∗∗4, 21. This guilt-by-association approach becomes more powerful when more drugs are profiled, as repetitive “chemogenomic” signatures, reflective of the general drug MoA, can be identified [18]. Yet, drug signatures are driven by pathways controlling the intracellular drug concentration as much as they depend on pathways related to drug MoA or its cytotoxic effects to the cell. Thus, machine-learning algorithms can be used to recognize the chemical-genetic interactions that are reflective of the drug's MoA. Although not yet used in such applications, Naïve Bayesian and Random Forest algorithms have been recently trained with chemical genetics data to predict drug–drug interactions ∗∗37, ∗∗38. Finally, although single-cell morphological profiling can be very powerful for MoA identification on its own ∗26, 39, it has not been used yet as a readout for large-scale chemical genetic screens in microbes. Small-scale screens do exist [40] and morphological profiling of wildtype cells has been combined only to a limited degree with growth-based chemical genetics [41]. In contrast, multi-parametric analysis of microscopy images is common for chemical genetic screens in cell lines, increasing the resolution for MoA identification [12]. Moving similar concepts to microbial chemical genetics is bound to improve our capacity for MoA identification.

## Chemical genetics in dissecting drug resistance

Chemical-genetic data are rich in insights into the routes that drugs use to come in and out of the cell, as well as into drug detoxification mechanisms, since both modulate the organism's ability to withstand the drug ∗∗2, 42, 43. Up to 12% of the genome confers multi-drug resistance in yeast [1], whereas only a few dozens of genes have similar pleiotropic roles in *Escherichia coli* [2], implying that prokaryotes have more diverse and/or redundant drug resistance mechanisms. Interestingly, when comparing chemical genetics data from deletion and overexpression libraries, it becomes apparent that many drug transporters and pumps are cryptic in bacteria: although they have the capacity to help the organism survive the drug treatment, they are not wired to sense the drug, remaining silent even in its presence. This non-optimal expression of drug transporters underlines the high capacity for intrinsic antibiotic resistance that microbes hold. Such resistance can be acquired by simply changing expression of non-optimally wired transport systems upon evolutionary pressure. Looking forward, with our increased capacity to construct and high-throughput profile mutant libraries, chemical genetics can be a great companion to Genome-Wide-Association-Studies or other types of sequenced-based computational models for prediction of antibiotic resistance potential within natural populations 44, 45.

**Figure 3:**
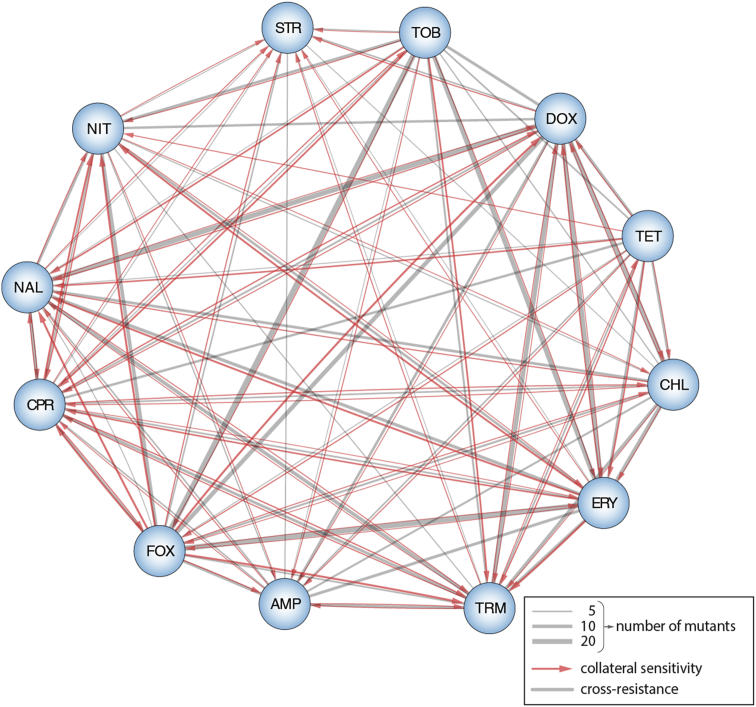
**Cross-resistance and collateral sensitivity maps using publsihed chemical genetics data**. Gray edges depict cross-resistance, i.e. gene-drug phenotypic events where mutants are either significantly more susceptible (negative) or more resistant (positive) to both drugs. Red edges depict directional collateral sensitivity- mutants which make cell more resistant to drug A, but more sensitive to drug B, or vice versa. Edge thickness denotes number of mutants. Data used to create network come from published chemical genetics data [2] and drugs selected based on overlap with previous cross-resistance studies 45, 46.

In addition to mapping resistance pathways, chemical genetics can be used to assess the level of cross-resistance and collateral sensitivity between drugs (Figure 3) – that is if mutations lead to resistance (or sensitivity) in both drugs, or make the cell more resistant to one drug but more sensitive to the other. Until now, these drug–drug relationships have been accessed by evolving resistance in bacterial populations to one drug, and testing if resistant clones are more sensitive/resistant to other drugs 46, 47, 48. Yet, this approach can survey only a limited number of potential resistance solutions, making it hard to assess if cross-resistance relationships between two drugs are monotonic or conserved across species. Also, the underlying genetic elements of cross-resistance remain unclear. Chemical genetics can overcome both obstacles, as they measure the contribution of every non-essential gene in the genome to resistance to different drugs (Figure 3). Evaluating cross-resistance patterns can facilitate our understanding of drug resistance, but also reveal paths to mitigate or even revert drug resistance [49].

## Chemical genetics in understanding and exploiting drug–drug interactions

**Figure 4:**
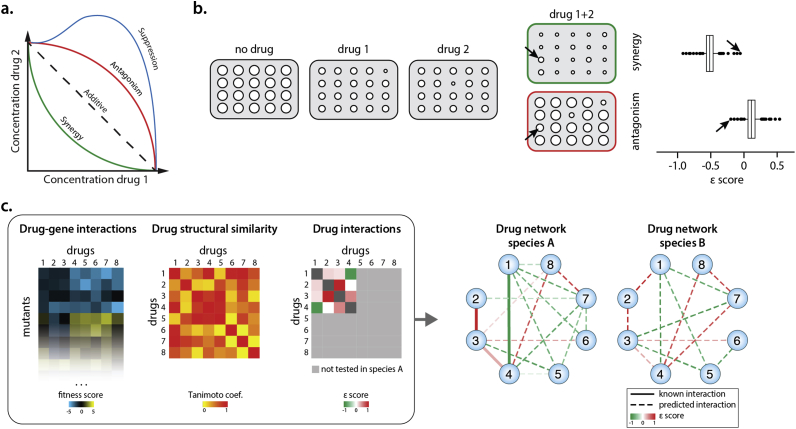
**Chemical genetics facilitate the mechanistic dissection and prediction of drug-drug interactions**. a) Isobologram illustrating different cases of drug interactions. Synergistic, antagonistic and suppressive interactions are represented as phenotypic deviations from the expected additive effect. b) Drug synergies or antagonisms can be profiled in a genome-wide library of mutants. A ε-score assessing the drug-drug interaction is calculated for every mutant, and corresponds to the difference of observed versus expected fitness in the presence of two drugs [48]. Although the vast majority of mutants exhibit a wildtype behavior, in some mutants the drug-drug interaction becomes neutral. These mutants are reflective of the molecular processes the drug interaction depends on. c) Chemical genetics data, drug structural features and previously known drug-drug interaction data can be integrated to predict a more complete drug-drug interaction network. Such networks can be extrapolated to phylogenetically related species.

Drugs when combined can synergize, antagonize or even mask each other's effects [50] (Figure 4a). Numerous recent efforts have aimed at mapping the combined effects of anti-infectives (antibiotics or antifungals) 51, 52 or that of combinations of anti-infectives with possible adjuvants 53, 54, 55 at larger scales. Yet the combinatorial space is enormous, the outcome can vary across species, and accurate assessment needs probing of combinations at multiple dose ratios, making systematic profiling very difficult. In clinics, drugs are combined to exploit synergistic activities, increasing the potency, widening the spectrum of action and reducing doses and side-effects of individual drugs. Most combinations used come from empirical knowledge and *ad hoc* testing, though drug interactions are often seen with fear by physicians and are characterized as “a common problem during drug treatment” in the EMA Guidelines [56]. The biggest current bottleneck for rational design of combinations with desired outcomes is our very limited understanding of drug–drug interactions, i.e. their underlying mechanisms and general principles.

Chemical genetics can be leveraged to shed light into drug–drug interactions, helping to dissect their underlying mechanisms ∗∗2, ∗∗57. In this case, drugs are profiled alone or in combination across the mutant library to identify the small fraction of genetic backgrounds in which the drug–drug interaction is not detectable anymore (Figure 4b). These genes that the interaction relies on are reflective of the molecular pathways and processes behind the interaction. Although drug–drug interactions are generally robust to genetic backgrounds, cells often require just single mutations to neutralize or even revert the interaction, e.g. make it from synergistic to antagonistic [57]. This begs the question of how conserved are drug–drug interactions within or across species, and what defines the degree of conservation.

At a second level, chemical genetics profiles have been used to predict drug–drug interactions outcomes. Drugs eliciting similar chemical genetic signatures not only often target the same cellular processes, but are also more likely to interact synergistically. This characteristic has been used to predict drug synergies 21, 58. Recently, more sophisticated approaches used an initial small set of ascertained drug combinations, chemical genetic screens, and sometimes additional information (genetic interactions, drug structural features) to train machine learning algorithms that can then better predict drug–drug interactions within the same species ∗∗37, ∗∗38 or even across species [38] (Figure 4c). Such machine-learning based probabilistic models will become even more powerful as chemical genetics data become available for more microbes [11], and as novel high-throughput quantitative information on drug-gene relationships emerge 59, ∗∗60.

## Discussion

Emerging drug resistance is becoming a major problem in many fields of medicine 61, 62, ∗63, with the situation in infectious diseases being utmost serious and posing an imminent threat to public health 64, 65. Development of new therapies is imperative, and formulation of new strategies to expedite this process is urgently needed. Expanding the chemical space probed to discover new bioactive molecules [66], reviving orphaned compounds [67], repurposing drugs [68] and leveraging off-target effects 69, ∗70 are some of the leading strategies towards new therapies. Chemical genetics can be instrumental in all these contexts by providing a systems-level view of the compound action, illuminating its effects on the targeted pathway, on routes the drug uses to come in and out of the cell and uncovering potential off-target effects [71]. This systemic view of the compound action is what gives chemical genetics strong descriptive and predictive power for the drug behavior, both at the MoA level and the level of drug–drug interactions. Combining chemical genetics with other types of orthogonal information on drug action ∗25, ∗26, ∗∗60, is one of the ways forward for building more accurate predictive models for drug MoA and drug–drug interactions. Moreover, linking chemical genetics to different quantitative, multi-parametric, macroscopic and single-cell readouts ∗20, 23, ∗25, ∗∗31, 72 will allow for profiling drugs that do not target cellular growth, and will more generally increase their resolution in dissecting the cellular role of drugs.
